# Influence of osteoporosis on the compressive properties of femoral cancellous bone and its dependence on various density parameters

**DOI:** 10.1038/s41598-021-92685-z

**Published:** 2021-06-24

**Authors:** F. Metzner, C. Neupetsch, J.-P. Fischer, W.-G. Drossel, C.-E. Heyde, S. Schleifenbaum

**Affiliations:** 1 Professorship for Adaptronics and Lightweight Design in Production, Institute for Machine Tools and Production Processes, Chemnitz University of Technology, Chemnitz, Germany; 2 Fraunhofer Institute for Machine Tools and Forming Technology, Dresden, Germany; 3 ZESBO - Center for Research On Musculoskeletal Systems, Department of Orthopaedic Surgery, Traumatology and Plastic Surgery, Leipzig University, Semmelweisstraße 14, 04103 Leipzig, Germany; 4 Department of Orthopedic Surgery, Traumatology and Plastic Surgery, Leipzig University, Leipzig, Germany

**Keywords:** Tissues, Biomedical engineering, Biomaterials, Structural materials

## Abstract

Data collection of mechanical parameters from compressive tests play a fundamental role in FE modelling of bone tissues or the developing and designing of bone implants, especially referring to osteoporosis or other forms of bone loss. A total of 43 cylindrical samples (Ø8 × 16 mm) were taken from 43 freshly frozen proximal femora using a tenon cutter. All femora underwent BMD measurement and additionally apparent- and relative- and bulk density (ρ_app_, ρ_r_, ρ_b_) were determined using samples bordering the compressive specimen on the proximal and distal regions. All samples were classified as "normal", "osteopenia" and "osteoporosis" based on the DEXA measurements. Distal apparent density was most suitable for predicting bone strength and BMD. One novel aspect is the examination of the plateau stress as it describes the stress at which the failure of spongious bone progresses. No significant differences in mechanical properties (compressive modulus E; compressive stress σ_max_ and plateau stress σ_p_) were found between osteopenic and osteoporotic bone. The results suggest that already in the case of a known osteopenia, actions should be taken as they are applied in the case of osteoporosis A review of the literature regarding extraction and testing methods illustrates the urgent need for standardized biomechanical compressive material testing.

## Introduction

The demands on implants are steadily increasing especially in regard to primary stability, i.e. the anchoring quality in the bone immediately after implantation. Particularly in case of pathological or age-related loss of bone strength such as osteoporosis, precise knowledge of the mechanical bone properties, based on clinical diagnostics is of crucial importance for selecting a suitable implant. Osteoporosis is a systemic disease associated with lower bone mass and degeneration of the microarchitecture of bone tissue leading to a reduction in mechanical strength and fracture resistance. It is usually diagnosed by a radiological examination of the lumbar spine and proximal femur using the dual-energy X-ray absorptiometry (DEXA). Based on measured bone mineral content (BMD [g/cm^2^]), the T-score is calculated as the deviation of the patient's BMD from a healthy control group. A distinction is made between normal (t > − 1), osteopenia (− 1 > t > − 2.5), and osteoporosis (t < − 2.5). Assigning the mechanical properties of a particular bone region to one of these standard categories may help to further develop numerical or physical bone models^1^.

The base of all bone models, whether numerically or artificially produced, is fundamental information about the mechanical properties of the bone, which must be determined experimentally using donor tissues. Human bone tissue exhibits a high variance of mechanical properties due to different loading situation and geometries. For this reason, the compliance with a uniform methodology for the experimental determination of mechanical bone properties is of utmost importance. A widely used method for determining the mechanical properties of human bone tissue is the compression test^2^. Despite the immense number of such examinations, no standardized protocol (ISO, ASTM, etc.) with specified process parameters has been established so far. For this reason, the data obtained from various studies are comparable only to a limited extent. An essential problem is the sparse information on how to take tissue samples. One aspect for standardization is the determination of the density parameters since they have great influence on bone strength^3,4^. The aim of the study is the description of similarities and differences in the methodological approach in the performance of compressive testing of human cancellous bone. Furthermore compressive tests will be conducted with the aim of referring the experimental mechanical and morphological parameters to clinical categories of osteoporosis.

## Materials and methods

### Specimen preparation technique

41 femoral heads from 26 human cadavers (21 females, 20 males) with mean age of 80.7 ± 10.9 years were obtained in fresh and anatomically unfixed condition. All body donors gave their informed and written consent to the donation of their bodies for teaching and research purposes while alive. Being part of the body donor program regulated by the Saxonian Death and Funeral Act of 1994 (third section, paragraph 18 item 8), institutional approval for the use of the post-mortem tissues of human body donors was obtained from the Institute of Anatomy, University of Leipzig. The authors declare that all experiments were conducted according to the principles of the Declaration of Helsinki. All bones underwent DEXA measurement for determination of bone mineral density (BMD). Based on the clinically determined T-scores all specimen were classified according to the definition specified by WHO as “normal” (N = 9), “osteopenia” (N = 20) and “osteoporosis” (N = 10). Due to inserted hip endoprosthesis two femurs could not be included in the study which leads to a total number of specimen of N = 39. All bones were stored fresh frozen at − 83 °C until further preparation.

**Figure 1:**
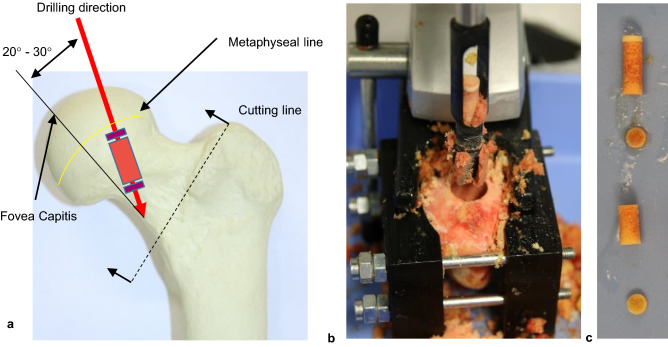
Specimen preparation: (**a**) shows the location of the samples within the available bone region. The drill penetrates into the diaphysis with an angle of 20°–30° in lateral direction, starting from the fovea capitis. In (**b**) a femoral head fixed in the clamping jaws can be seen immediately after drilling. The fovea capitis is visible in (**b**) as a yellow and dark red spot below the drill hole. The already cut drill core can be seen in (**c**). The parts are arranged from proximal (top) to distal (bottom).

The femoral heads were separated from the remaining bone according to the procedure for inserting a hip endoprosthesis. The separation line runs through the femoral neck one to two centimetres distally to the femoral head. The removal of the drill cores was carried out with a tenon cutter (FAMAG Series 1616, FAMAG-Werkzeugfabrik GmbH & Co. KG, Remscheid, Germany) and a stationary drilling machine (model PBD 40; Robert Bosch GmbH Power Tools, Leinfelden-Echterdingen, Germany) in superior-inferior direction along the trabecular alignment in the femoral neck as displayed in Fig. 1a.

**Figure 2:**
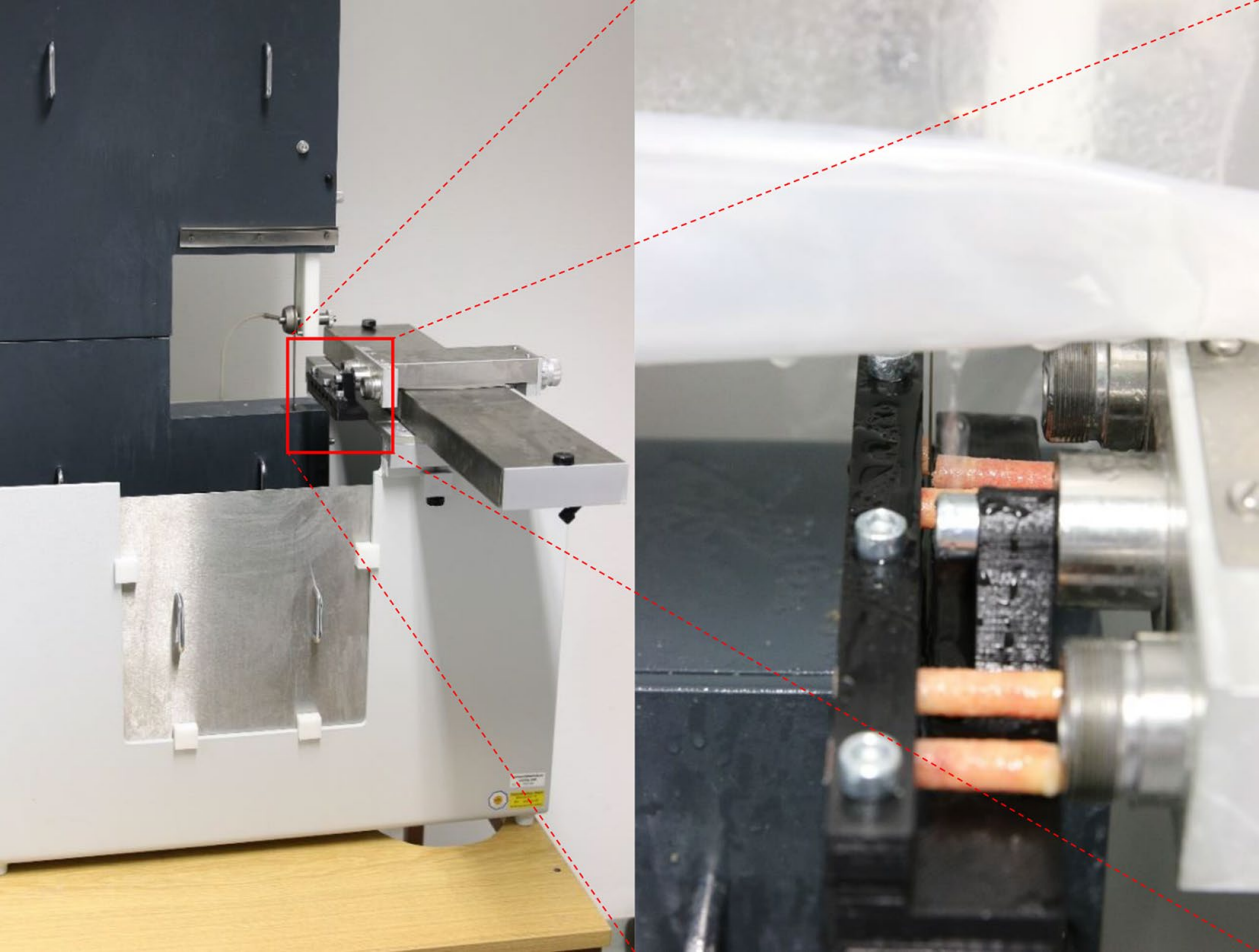
Self constructed and 3D printed clamping jaws are mounted at the parallel guidance of the band saw. The mounted section of the drill core is later used as mechanical specimen. Sawing was carried out under permanent water irrigation.

To align the drilling direction with the main trabecular orientation the bones were clamped in self-constructed and 3d printed jaws (see Fig. 1b). There were conical cut-outs on the inside of the jaws to prevent the bones from slipping. The still frozen femoral heads were placed on a lying jaw by hand. Using the tendon attachment of the ligamentum caput femoris as guidance the head was inserted with the drilling direction parallel to the frontal plane. Afterwards, the bone was tilted so that the drilling direction (see Fig. 1a) was orthogonal to the upper edge of the clamping jaw. The samples are cut to size with a diamond band saw (EXAKT 310, EXAKT Advanced Technologies GmbH, Norderstedt) to ensure parallel end faces. The drill cores are fixed again in 3d printed jaws (see Fig. 2 right) during the sawing process. Orthogonal end faces could be provided due to fixation of the jaws to the parallel guide in the band saw. The drill cores were clamped in the areas, which were later mechanically tested, in order to avoid misalignment due to re-clamping. A sample geometry of Ø 8 × 16 mm is used for all mechanical specimens. In previous studies, as seen in Table 3, a diameter of 8 mm is commonly used as it is wide enough to include enough trabecular connections^5^. The length ratio of diameter to length of 1:2 is specified and recommended in DIN ISO 50134^6^ an therefore was implemented in the present study.

Two slices of 2 mm thickness were cut from the drill core for density determination (see Fig. 1c). Each of the slices originated from the areas bordering the mechanical specimen proximally/distally. These slices were defatted by a 5-day ethanol storage at 4 °C followed by three days storage in NaCl-solution (0.9%) at 21 °C. The following density parameters of were then measured according to the Archimedean principle with ethanol as density medium using a precision scale with integrated density kit (Mettler-Toledo GmbH, Gießen, Germany, Model ML303T/00). Apparent density ρ_app_ was determined from bone mass and sample volume. Bulk density ρ_b_ is calculated from bone mass and bone volume. Additionally the relative density ρ_r_ was calculated by the ratio of defatted bone volume and the sample volume. The nominal geometry of all samples is measured with a calliper gauge. Sample length and diameter of the discs is determined at one measuring point. The diameter of the specimen used for pressure tests is determined as the average of three measuring points. In between all processing steps the samples are stored in physiological saline solution to avoid dehydration.

### Mechanical testing

The compression tests were carried out on a servo-electric single-axis testing machine (INSTRON GmbH, Darmstadt, Germany). The force was recorded by a force transducer (measuring range 2 kN) and the strain measurement was performed via the machine traverse. Machine compliance was not adjusted. The force was applied via two plane-parallel anvils. Before each test, the test surfaces were cleaned and moistened with Ringer's solution to minimize friction on the contact surfaces. The testing procedure starts with the application of the preload of 10 N before going through a hysteresis loop for pre-conditioning according to^6^. Therefore the test force is increased up to an equivalent stress value of σ_1_ = 3.2 MPa and subsequently released to σ_2_ = 1.6 MPa. After passing through the hysteresis loop once the anvil continues and compresses the specimen until an total distance of 12 mm is reached or the measuring range of the force transducer is exceeded. The reversal points of the hysteresis loop result from the mean plateau stress of a pre-test series with n = 5 specimens (σ_1_ = 0.2 * σ_p_; σ_2_ = 0.4 * σ_p_^6^). According to^6^ plateau stress σ_p_ is defined as the mean value of the stresses between ε = 20% and ε = 40%. This value marks the average stress of failure progression after initial structural collapse. Up to a deformation of 10%, the test is performed at a test speed of v_1_ = 0.016 mm/s, then v_2_ = 0.16 mm/s for a sufficiently high sampling rate in the linear range to ensure accurate values determining the compressive modulus. Then the test speed is increased to v_2_ = 0.16 mm/s in order to save time in the plateau range. From the measured values, the parameters plateau stress σ_p_, compressive stress σ_max_ and compressive modulus E are determined. The compressive stress σ_max_ is the stress value of the first maximum in the curve which marks the initial structural failure. In literature this value is often called ultimate stress. E is determined using the mathematical slope formula for linear functions from the reversal points of the hysteresis. In case samples fail while passing through the hysteresis, the measurement is aborted after reaching σ_max_. Then E is determined from the maximum gradient of the slope and no σ_p_ is determined. All measures were carried out within one day to avoid storage influences.

### Statistical analyses

Descriptive statistics were examined using IBM SPSS Statistics 24 (IBM Corporation, Armonk, NY, USA). T-tests for independent samples were carried out between 5 different pairs of groups (male vs. female; left vs. right; normal vs. osteoporosis; normal vs. osteopenia; osteopenia vs. osteoporosis) for all measured structural and mechanical parameter. Statistical significance is defined with p < 0.05. Simple linear regression was used to determine relationships among density parameters. Regression between mechanical parameters and the density parameters was carried out by a power function.

## Results

**Figure 3:**
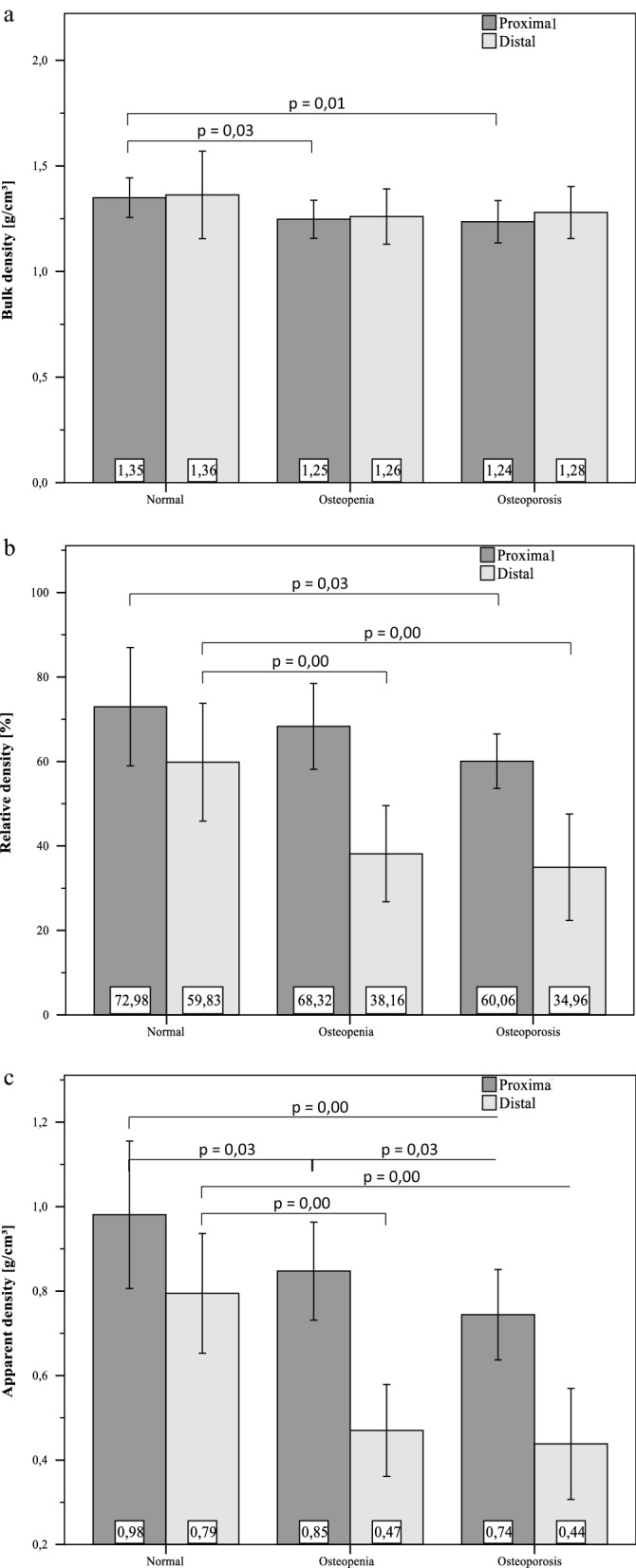
Mean density values of the density samples collected proximally and distally oriented to the mechanical specimen. The data grouped regarding WHO classification for osteoporosis based on BMD measurements of each donor bone. Bulk density ρ_b_ (**a**) stays roughly constant with low differences between sites. Apparent density ρ_app_ (**b**) and relative density ρ_r_ (**c**) show both great differences both between sites and groups. all significant differences are marked with their specific p-value from independent t-tests.

The mechanical samples (n = 40) have a diameter of d = 8.12 mm ± 0.07 mm, a length of L = 15.98 ± 0.06 mm and the density samples have a length of L_S_ = 1.98 mm ± 0.05 mm. The mean BMD of 0.69 g/cm^2^ lead to an average T-score of − 1.69. Average values as well as their standard deviations are displayed in Fig. 3a–c for each distal and proximal bone slices, grouped into the previously defined stages of osteoporosis. The differences in the density values between the proximal and distal slices is around 0.02 g/cm^3^ for ρ_b_, 30% for ρ_r_ and 0.3 g/cm^3^ for ρ_app_. Relative and apparent densities of the distal slices are clearly lower than the proximal relatives. While the proximal bulk density falls significantly from “normal” to “osteopenia” (p = 0.03) and from “normal” to “osteoporosis” (p = 0.01), there are is no significant difference between “osteopenia” and osteoporosis” (p = 0.76). No significant differences were found between the distal bulk densities. The proximal relative density of “normal” specimen is not significantly higher than “osteopenia” (p = 0.34) but is significantly higher than “osteoporosis” (p = 0.03). The distal relative density drops significantly from normal “normal” to “osteopenia” (p = 0.00) and from “normal” to “osteoporosis” (p = 0.00), but not from “osteopenia” to “osteoporosis” (p = 0.50). An almost similar pattern is seen for the apparent density. The proximal apparent density drops significantly from “normal” to “osteopenia” (p = 0.03), from “normal” to “osteoporosis” (p = 0.00) and also from “osteopenia” to “osteoporosis” (p = 0.03). The distal apparent density drops significantly from “normal” to “osteopenia” (p = 0.00), from “normal” to “osteoporosis” (p = 0.00) but not from “osteopenia” to “osteoporosis” (p = 0.49).

Table 1 shows the R^2^ values of simple linear regression analyses. The clinical parameters BMD and T-score are used as independent variables for explanation of the variances in each determined density measures. Highest R^2^ values were found for the distal apparent density of the distal slices as displayed in Table 1. The model quality worked slightly better with the BMD as independent variable.

**Table 1:** R^2^ values of linear regression analysis by plotting each measured density values over T-score and BMD. Significant models are marked with *.

R^2^	T-score	BMD
ρ_b,proximal_	0.182*	0.160*
ρ_b,distal_	0.068	0.044
ρ_r,proximal_	0.105*	0.082
ρ_r,distal_	0.327*	0.384*
ρ_app,proximal_	0.244*	0.203*
ρ_app,distal_	0.493*	0.543*

**Figure 4:**
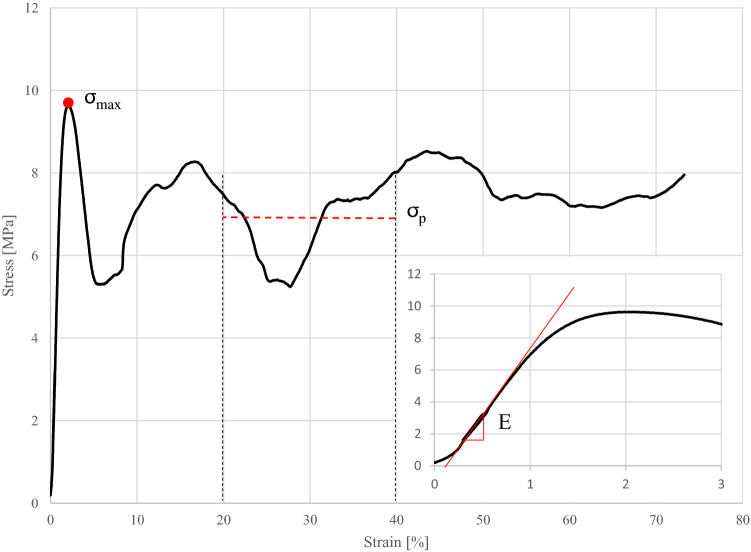
A typical stress–strain curve of one measured specimen is displayed. After a little toe region due to misalignment of the anvils and the specimen endfaces, the following linear region defines the compressive Modulus E and is nicely represented by the turning points of the hysteresis loop. After the first maximum σ_max_ which marks the initial structural failure the stress drops at first and continues with irregular swelling. Despite the high swelling amplitude the plateau stress agrees well with the average stress in the areas of high strains.

Figure 4 shows a typical stress–strain curve with a close-up on the initial force application. There is a toe region located at the beginning followed up by the hysteresis loop. The red line marks the turning points which defines the compressive modulus and fits nicely with the maximum slope of the linear region of the curve. Additionally, there was no visible plastic deformation after preconditioning.

**Figure 5:**
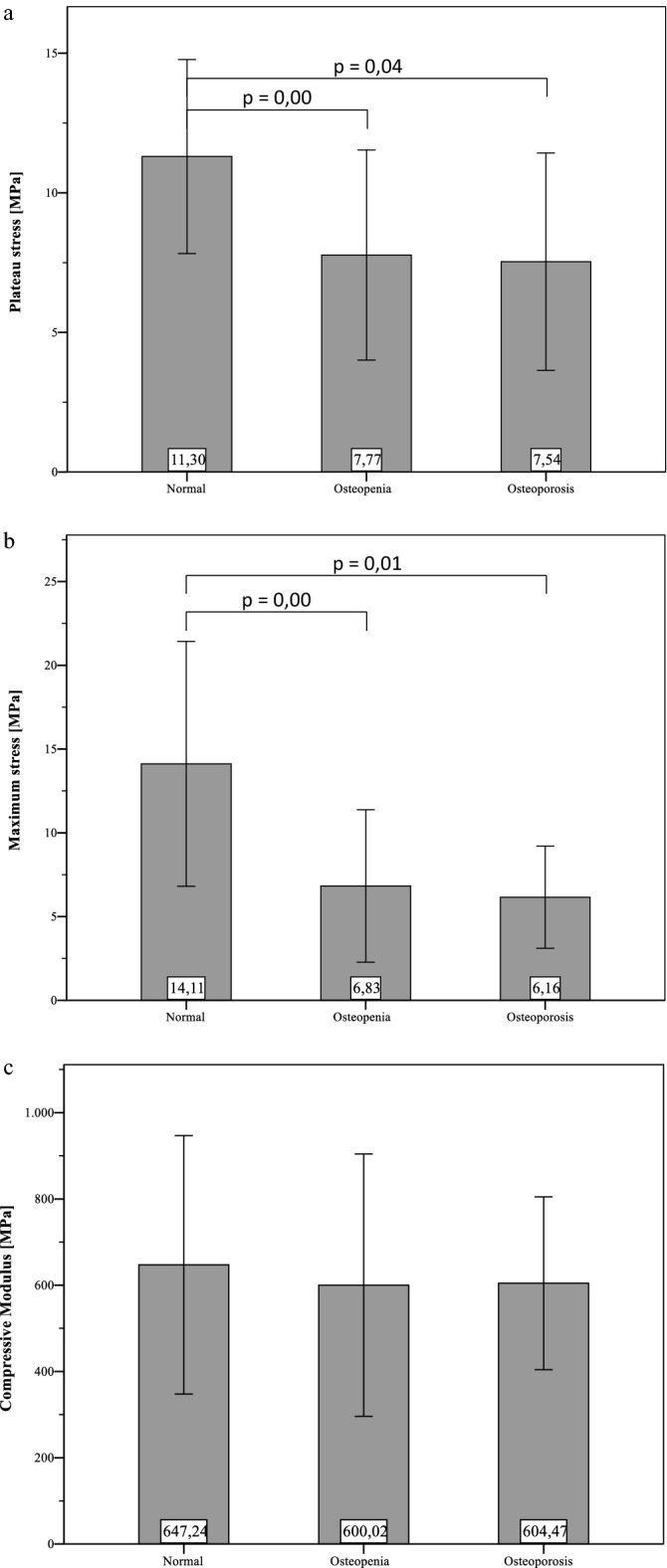
Mean values (with standard deviation) of the determined mechanical properties, grouped regarding WHO classification for osteoporosis based on BMD measurements of each donor bone. There are no significant differences in all mechanical properties (σ_p_, σ_max_, E) between “osteopenia” and “osteoporosis”. Significant differences within presented groups are marked with their specific p-value from independent t-tests.

All measured mechanical parameters are grouped similar to the previously displayed density measures as displayed in Fig. 5a–c. σ_max_ of “normal” bones (14.1 ± 7.3 MPa) is significantly higher (p = 0.00) than bones with “osteopenia” (6.8 ± 4.5 MPa, p = 0.00) and as bones with “osteoporosis” (6.1 ± 3.1 MPa, p = 0.01). The plateau stresses show comparable results. σ_p_ of “normal” bones (11.3 ± 3.5 MPa) is significantly higher than bones with “osteopenia” (7.8 ± 3.8 MPa; p = 0.00) and “osteoporosis” (7.5 ± 3.9 MPa; p = 0.04). The average difference of σ_p_ between “osteopenia” and “osteoporosis” is not significant (p = 0.9). There were no significant differences between the moduli of each group.

**Figure 6:**
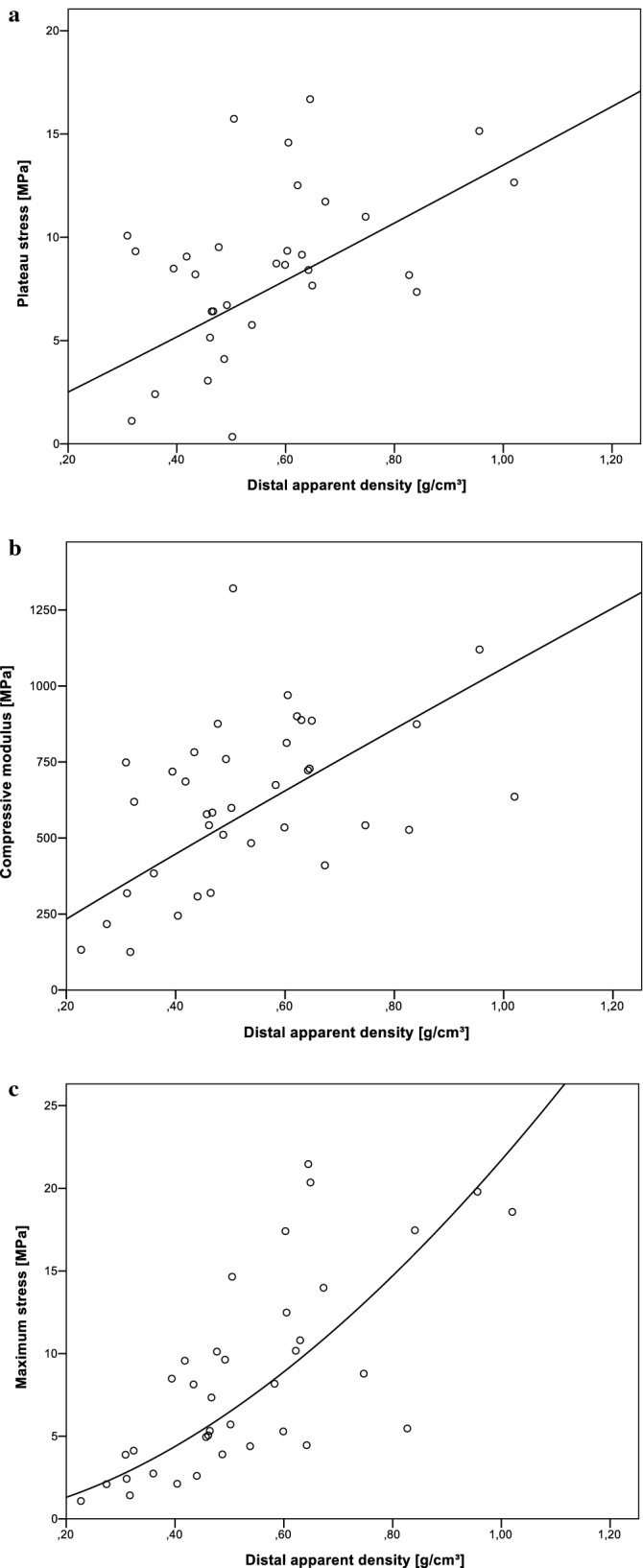
Mechanical properties plotted as power function over the distal apparent density showed best models according to R^2^ values (s. Table 2).

Regression analysis of the mechanical parameters (σ_max_, σ_p_, and E) with the all available density measures using a power function demonstrate that the distal apparent density shows highest R^2^ values as seen in Table 2. Figure 6a–c show the previously mentioned plots of each mechanical parameter over the distal apparent density. The specimen of female donors hat significantly lower T-score (P = 0.00), BMD (P = 0.00), ρ_r,distal_ (P = 0.01), ρ_app,proximal_ (P = 0.02) and ρ_app,distal_ (P = 0.00) than the ones from male donors.

**Table 2:** R^2^ values of regression analysis using power function. All three mechanical parameters can be best explained by distal apparent density. Significant models are marked with *.

R^2^	σ_p_	σ_max_	E
ρ_b,proximal_	0.041	0.127*	0.033
ρ_b,distal_	0.048	0.090	0.069
ρ_r,proximal_	0.114	0.002	0.005
ρ_r,distal_	0.079	0.392*	0.226*
ρ_app,proximal_	0.198*	0.046	0.000
ρ_app,distal_	0.161*	0.598*	0.360*
BMD	0.042	0.099	0.009

## Discussion

Cylindrical samples were collected from the femoral neck with a drill along the main trabecular orientation. Directly distal to the epiphyseal line, three samples were cut from each drill core. A cylindrical specimen with a length to diameter ratio of 2:1 for mechanical testing and adjacent 2 mm discs were collected proximally and distally for examination of morphological parameters.

All density measures of both distal and proximal slices decreased with progressing osteoporosis. This is an obvious result as it is known that osteoporosis leads to bone loss and downgrade of trabecular architecture^1^. But only ρ_r,proximal_ and ρ_app,proximal_ show a significant difference between osteoporotic and osteopenic specimen. There is just a slight difference in the bulk densities of about 0.5 g/cm^3^ between the specimen location which underlines the effectiveness of the defatting method as the bone density should be the same over each drill core. By regression analysis using power function seen in Table 2 it was shown that the mechanical properties can be best explained by the apparent density of the distal slices. About 60% of the variance in σ_max_ can be explained by ρ_app,distal_. It was found that the effectiveness of predicting the mechanical properties works best using the distal slices, which have the lower density values and therefore this region predicts some kind of predetermined breaking area within the mechanical specimen. The reason may simply be that areas with a low density will break earlier than denser areas as described by^7^. The high explanatory accuracy of the mechanical parameters using separate discs works better than relating it to the outcome of DEXA measurement. The undescribed portions of data may be due to misalignment of the specimen axis and the main trabecular orientation. So, if there is found a simple way for determining the main trabecular orientation on the extracted specimens, the present method of density measurement may be an alternative for µCT-scanning.

Also, the bone marrow may not be completely removed by the baths in ethanol and saline solution. Apparent and relative density rise therefore with increasing bulk-volume. This could be an explanation why the distal apparent density works best for prediction of BMD or mechanical parameters as seen in Tables 1 and 2. We assume that ρ_app_ is less susceptible to measurement errors than the other determined density parameters. If the bone marrow is not completely removed during defatting, the mass fraction of the marrow still contained in the sample is probably significantly smaller than the volume fraction of the said marrow. For the geometric density, the sample mass is related to the total sample volume, so only the mass and not the volume of the undissolved bone marrow is included. Therefore the same methodical error is taken less into account.

Using separate test specimens for density determination, no further processing of the mechanically tested specimens takes place apart from the extraction process. For that, the reducing influence of bone marrow on the compressive stress as said by^8^ was taken into account. The defatting of the bone is necessary for determining the bone density according to the Archimedean principle for the determination of bone mass. The defatting process is facilitated by the small dimensions of the density samples and the associated short diffusion paths.

By grouping the samples into the stages of osteoporosis it was shown that bone strength (σ_max_, σ_p_) generally decreases as osteoporosis progresses, whereas the modulus remains relatively constant at about 600 MPa for each group. The largest jump is visible in both maximum stress and plateau stress from "normal" to "osteopenic" bone. Although the samples classified as osteoporotic and osteopenic do not show significant differences. These results suggest that already in the case of a known osteopenia, actions should be taken as they are applied in the case of osteoporosis (e.g. cementation of implants, imaging techniques). The predictive power of the BMD decreases with younger patients^9^. Since the body donors from which the samples were obtained were on average very old, this question should be pursued further and the sample should be expanded accordingly.

It is interesting to note that the maximum stress of the "normal" specimens is on average about 2–3 MPa above the plateau stress. However, the plateau stress of the osteopenic and osteoporotic samples is about 1 MPa higher than the maximum stress. Thus, the initial failure stress of healthy bone is very high, while the forces required for progression of failure are comparatively low. This could be helpful for evaluating the primary stability of implants which are anchored mainly in trabecular bone, since e.g. the surrounding tissue initially fails while implanting bone screws therefore loosening is merely failure progression. It must also be taken into account that the test speed is increased by a factor of 10 after the maximum stress is exceeded. Consequently, the plateau stress could be underestimated due to the influence of the loading speed on the material stiffness. Carter and Hayes^10^ reported, that energy absorption increases at strain rates of minimum 10.0 per second. Also they found out, that both modulus and strength increase proportional to strain rate with a power of 0.06. Accordingly, this means that the tenfold increase in speed leads to an increase in modulus and strength by a factor of about 1.16. The extent to which this affects plateau stress cannot be precisely stated at this point. Though it is assumed that there is an influence, which is relatively small.

The plateau stress as it is found here has only been determined in a few studies in a comparable way. Halgrin et al.^8^ determined the plateau stress (σ_mean_) as the mean stress between the compressive stress and an elongation value of 60%. They investigated cancellous bone from the bovine ribs. A very long plateau area was observed with stress values slightly below the compressive limit. Although the absolute stiffness values cannot be directly compared, the present study showed very similar post-yield behaviour. Fhyrie and Schaffler^15^ examined the post-yield behaviour up to an elongation of 15%. They determined the minimum stress that occurs after failure as a characteristic value. Interestingly, they found a significant correlation between stiffness and the minimum stress after failure.

In contrast to the post-yield behaviour of bovine bone examined by^8^, the present study recorded large fluctuations in the highly plastic range. Some curves show irregular swelling behaviour, which indicates an inhomogeneous structure within the samples. This could be explained as follows. As the weak zones collapse, the stress curve decreases. If the now compacted material hits a stronger zone, the stress rises again until next failure. This behaviour is repeated several times until the material is finally compacted.

The measured maximum stresses are even higher than the values determined in^11^ for the identical bone region (yield strength: 6.7 MPa; compressive modulus 263 MPa). Nazarian et al.^11^ used brass plates glued to the front sides of the specimens in order to eliminate transverse forces and to prevent buckling of the trabeculae at the interface. Also a ball joint was introduced to compensate for parallelism deviations, thus preventing underestimation of the bone strength in the axial direction. With this measurement setup the determined modulus should be higher than the moduli of the present study as the authors tested the same bone region and the mean age of the donors was the same on average. Additionally Nazarian et al. used an embedding system for prevention of end artefacts as described by^12^.

Ciarelli et al.^13^, Morgan et al.^14^ and Perilli et al.^15^ tested cancellous bone from the identical bone region as the present study and obtained mean compressive stresses of around 15 MPa and matched very good with the ”normal” group. With an average compressive modulus of 1100 MPa^13^ measured almost twice as high values as the present study. The compressive Modulus determined by^14^ where around 5 times higher. The present stiffness E should be interpreted with cation as the machine compliance was not taken into account. With the approximately known underestimation of the stiffness due to direct force application of around 40% mentioned by^16^ the real compressive modulus could be around 700 GPa or higher. The differences of the presented stiffness values compared to the literature can best be explained by the use of direct implantation of the testing force in the specimen. This leads to end-artefacts because the trabecular ends are not supported. The anvils were lubricated before testing, but end artefacts are the main explanation of the comparably low stiffness values. Nevertheless it is possible to point out differences within each group in terms of osteoporosis. One potential limitation of the present study is that it does not address or improve evaluation and assessment of fragility fractures. They occur in vivo on whole bones, whereas in this work isolated spongious bone specimen where examined. The aim was to provide an overview of previous methods for determining the mechanical parameters of bones in uniaxial compression tests as well as comparing these to clinical outcomes like T-score, something that may or may not be clinically relevant as far as fragility fractures are concerned.

Due to the large differences in the mechanical properties of the identical anatomical region between different studies, an overview of different sampling and testing methods is shown below. Table 3 shows the methodological approach of a large number of studies arranged according to the bone region examined. Special attention was put on the method of specimen collection, specimen geometry, test protocol and recorded test parameters rather than on actually recorded results. This intends to illustrate the large the discrepancy in the methodology of the current state of research. The listed studies represent only a fraction of all studies that investigate the compression behaviour of cancellous bone. Only those studies were selected which determine human bone properties in the uniaxial compression test.

**Table 3:** Survey of methodological designs of published literature regarding the compressive testing of human cancellous bone categorized over examined body parts.

Region	Author	Specimen geometry				Specimen geometry	Testing protocol			Parameters	Scope of study (donors; specimen)
		Tools	Bone alignment	Specimen alignment	Density measurement		Preconditioning (Number of Cycles; upper Limit)	Force initiation	End of testing		
Proximal femur	Present study	T; B	C	SI	A	Cylinder (Ø 6 × 16 mm)	1; from pre study	D	ε = 70%	E, σ_max_, σ_p_	5; 10
	Ciarelli, Fyhrie et al.^13^	B	I	SI	μCT	Cube (8 × 8 mm)	10; ε = 0.4%	D	ε = 15%	E, σ_max_	58; 58
	Martens, van Audekercke et al.^17^	B; T	I; E	SI,ML,AP	–	Cylinder (Ø 8 × 8 mm)	–	D	ε = 15%	E, σ_max_	20; 519
	Brown & Ferguson ^18^	B	–	SI,ML,AP	–	Cube (5 × 5 mm)	–	D	F	E, σ_y_	–; 800
	Chevalier et al.^19^	B; T	–	SI	μCT, A	Cylinder (8 × 12 mm)	10; ε = 0.35%	ET	F	E, σ_y_	3; 6
	Goulet et al.^20^	M	E	SI,ML,AP	μCT, A	Cube (8 × 8 mm)	10; σ_y_/2	D	F	E, σ_max_	4; 12
	Nazarian et al.^11^	T	I	7 directions	μCT	Cylinder (Ø5 × 10 mm)	7; –	ET	F	E, σ_y_ ,ε_y_	7; 47
	Matsuura et al.^21^	B; T	I	SI	μCT	Cylinder (Ø8 × 12 mm)	12; ε = 0.35%	E	ε = 6%	E, σ_y_, σ_max_	185;146
	Morgan et al.^14^	T	I	SI	A	Cylinder (Ø8 × 20 mm)	3; ε = 0.1% and 10; ε = 0.3%	E and D	ε = 0.5%	E, σ_y_, ε_y_	42; 33
	Homminga et al.^22^	B	I	SI	μCT	Cube (5 × 5 × 5 mm)	10; ε = 0.4%	D	ε = 15%	E, σ_y_ ε_y_	–;30
	Perilli et al.^15^	B; M	I	SI	μCT	Cylinder (Ø 10 × 26 mm)	–	ET	F	σ_max_	–; 50
	Öhman et al.^23^	B; M	I	SI, specified offset (20°)	μCT	Cylinder (Ø 10 × 26 mm)	–	ET	F	E, σ_max_	–; 10
	Mazurkiewitz ^24^	B; T *	–	SI	μCT	Cylinder (Ø 10 × 8.5 mm)	5; ε = 0.65%	D	F	σ_max_	–;30
	Tassani et. al. ^7^	M	I	SI	μCT	Cylinder (Ø 10 × 20 mm) ?	–	ET	F	σ_max_	–, 25
	Schoenfeld et al.^25^	B; T	–	SI, ML	?	Cylinder (Ø 4.5 × 9.5 mm)	–	D	F	σ_max_	–; 30
Distal femur	Goulet et al.^20^	M	E	SI	μCT, A	Cube (8 × 8 mm)	10; σ_y_/2	D	F	E, σ_max_	4; 39
Proximal tibia	Goulet et al.^20^	M	E	SI	μCT, A	Cube (8 × 8 mm)	10; σ_y_/2	D	F	E, σ_max_	4; 27
Vertebrae	Goulet et al.^20^	M	E	SI	μCT, A	Cube (8 × 8 mm)	10; σ_y_/2	D	F	E, σ_max_	4; 4
	Keaveney et al.^12^	B; T; L	I	SI	A	Cylinder (Ø 8 × 35 mm)	10; ε = 0.3%	E	ε = 3%	E,	15; 15
	Keaveney et al.^26^	T;	–	SI	A	Cylinder (Ø 8.3 × 20…30 mm)	Varying preconditioning	E	ε = 4%	E, σ_max_	15; 50
	Wegrzyn et al.^27^		I	SI,AP	μCT	Whole vetebral body	10; 50–100 N	E	F	Failure load, compressive stiffness	21;21
	Matsuura et al.^21^	B; T	I	SI	μCT	Cylinder (Ø8 × 12 mm)	12; ε = 0.35%	E	ε = 6%	E, σ_y_, σ_max_	185; 307
Pelvis	Dalstra et al.^28^	B	CS	SI,ML,AP		Cube (6.5 × 6.5 mm)	5–10; ε = 0.8%	D	–	E	2; 57
Calcaneus	Matsuura et al.^21^	B; T	I	ML	μCT	Cylinder (Ø 8 × 12 mm)	12; ε = 0.35%	E	ε = 6%	E, σ_y_, σ_max_	185;128
Radius	Matsuura et al.^21^	B; T	I	ML	μCT	Cylinder (Ø8 × 12 mm)	12; ε = 0.35%	E	ε = 6%	E, σ_y_, σ_max_	185;162

*A* Archimedic principle, *AP* anterior–posterior, *B* band saw, *C* clamps, *CS* coordinate-system, *D* direct contact, *E* embedding, *ET* endcap-technique, *F* failure of material, *I* imaging-methods, *L* lathe, *M* milling, *ML* medial–lateral, *SI* superior–inferior, *T* trephine, *σ*_*max*_ first maximum/ultimate stress, *σ*_*y*_ yield stress.

When specimen are taken from bone parts, mechanical parameters are usually determined along the predominant trabecular alignment. Due to the strongly anisotropic material behaviour of the cancellous bone, the precise alignment of the bone tissue is of highest priority, since this step is fundamental for the informative value of the entire study.

Unfortunately, the extraction method and positioning of tissue is often described vaguely. In addition, there are numerous methodical approaches as displayed in Table 3. A diamond coated band saw is often used to cut discs from the bone, from which the drill cores are then removed perpendicular to the cutting plane with a trephine drill^17,19,21,24,25^. Some determine the position of the section planes by means of trabecular alignment using imaging techniques such as contact X-rays or CT^17,21^. Another method for aligning bone tissue is to embed the bones or bone segments in cast resin or plaster^20,29^. Regardless of the procedure, during all of the above methods a bony object must be aligned manually at some point. The jaws used in this study for clamping and aligning bone tissue, in conjunction with the use of imaging techniques, represent a fast and flexible alternative to embedding, since the curing times involved are very time-consuming. Still one limitation is the alignment of the femoral heads in the clamping device, since the course of the trabecula is not exactly visible from the outside. At this point some improvements need to be done in future experiments.

Removing drill cores with a tenon cutter enables working without additional cooling medium due to allow heat accumulation. Normally, tenon cutter are used to create wooden plugs with fixed external dimensions which is very useful for mechanical testing. Furthermore, the used tool offers the opportunity to remove very long drill cores in comparison to ceramic drills. For evaluation purposes the drill cores were extracted from frozen bone. After extraction the cores showed no outer damages. Apart from that, the marrow was still in a frozen state and therefore supported the bone structure in between while drilling. Trephines or diamond coated drill cores are widely used for extracting cylindrical samples^11,12,17,19,21,24,25^. The advantages of this tool are clean cutting surfaces and a small outer radius, which allows more specimens to be extracted from the limited space of a bone part. However, cooling is required during drilling to prevent tissue damage possibly caused by frictional heating. In addition, bone chip transport could prove to be problematic, since it can impair cooling at great drilling depths. The used tenon cutter provides an improved chip transport and a lower number of cutting edges resulting in less heat development. This eliminates the need for cooling during drilling. Furthermore, the tenon cutter enables the extraction of longer drill cores compared to the trephine.

Cutting the drill cores to size using the parallel guiding system of the band saw as well as a 3D printed clamping device leads to precise plane-parallel cutting end faces, which ensure uniform and centric force application into the sample. Still, small but unavoidable misalignment errors of the anvils led to minor initial toe regions.

The revised literature (see Table 3) contains mostly cube-shaped and cylindrical specimens with a width or diameter between 5 and 10 mm. The ratio of sample length to diameter varies between 1 and 2. This trend in the literature is based on the work of Linde et al.^30^, who investigated the effect of sample geometry on mechanical behaviour and recommended a diameter of 7.5 mm for cylindrical samples. In the present study we used cylindrical specimens with a diameter of 8 mm. This diameter was chosen because it is most frequently found in literature (see Table 3) and additionally very close to diameter suggested by^30^.

The mechanical properties of cancellous bone strongly depend on density^4,31–35^, hence density determination is an essential part of biomechanical testing of bone tissue. The most frequently determined parameters include apparent density and bone volume fraction. The Archimedean principle and the high-resolution μCT are the methods most widely used for this purpose as presented in Table 3. While density determination using the Archimedean principle appears to be quick and easy, the bone marrow must first be removed from the sample in a preceding step if information about the bone volume content is needed. There are various solutions for this process step, such as blowing out with an air jet, chemical dissolving, the use of ultrasound baths or the combination of several approaches^19,20,36^. However, as the sample size increases and the pore size of the spaces decreases, this process becomes more difficult or more complex to perform due to larger diffusion paths.

The determination of bone density using μCT is contactless, but requires a lot of effort. The accuracy of this method depends not only on the resolution but also on the determination and validation of a threshold value during segmentation^19^. A major advantage of μCT analysis of extracted spongious bone specimen is the precise determination of structural parameters like trabecular thickness or trabecular connectivity. However, this method is very time and cost consuming compared to the much simpler submersion techniques. Another option would be QCT, as it can accurately determine the apparente density of the bone, and this value is well suited to predict the mechanical properties of cancellous bone. In clinical practice, this method is used less frequently than DXA due to the high radiation exposure to patients. For biomechanical analyses of bone, QCT could well be used instead of μCT.

The way in which the test force is transferred to the specimen has a great influence on the mechanical properties. In older studies^18,20,25^ due to simplicity the test force was applied directly from the plane-parallel anvils into the specimen. Therefore the unsupported trabecular ends at the end faces are subjected to undesirable bending forces which lead to increased strain in the boundary areas of the specimen. As a consequence the stiffness of the bone is underestimated by around 40%^16^. This systematic error can be reduced by embedding the trabecular ends using cyanoacrylate or casting resins^12,30^. Another method is to glue thin metal plates to the sample ends with cyanoacrylate^11,19,29^, as the adhesive hardens quickly and the plates ensure that the adhesive is not subjected to any transverse forces that could loosen the bond while testing. For a comparison of the examined groups a consistent method was carried out. But care must be taken when comparing the compressive modulus within other studies.

Before the actual destructive test, several loading and unloading cycles are usually performed. The advantages of this preconditioning are the setting of the material to compensate for alignment errors and the exclusion of possibly damaged specimens^11,12^. Usually approx. 10 cycles are run between ε = 0% (lower limit) and ε = 0.35% (upper limit). In the present study one hysteresis loop was performed as preconditioning. The limits of the hysteresis were determined in a pre-test as it is prescribed in^6^.

## Conclusion

For the fixation of the femoral heads during specimen extraction, generatively manufactured clamping jaws proved to be an effective instrument that enables fast and simple handling. Additionally it is cost-efficient in production, due to a more and more widespread use of desktop 3D-printers. The determination of bone density using separate discs from areas bordering the mechanical specimens worked well for the description of plateau and compressive stress. Consequently, a cost- and labour-intensive μCT does not have to be carried out and the mechanical specimens remain unaffected and can be tested up to very large deformation ranges. The compact review of the extraction and testing methods from the literature illustrates the urgent need for standards regarding uniaxial compressive testing of human bone.

When comparing the different stages of osteoporosis based on the DEXA measurements, a general decrease in density and bone strength was observed with more pronounced disease progression. ρ_app,distal_ was most suitable for predicting bone strength. No significant differences in mechanical properties (E, σ_max_, σ_p_) were found between osteopenic and osteoporotic bone.

The prediction of bone strength in clinical environment should be considered in a very sensitive way. The results suggest that already in the case of a known osteopenia, actions should be taken as they are applied in the case of osteoporosis (e.g. cementation of implants, imaging techniques).

## Supplementary Information

Supplementary Information.
