# Supplementary material for: Influence of osteoporosis on the compressive properties of femoral cancellous bone and its dependence on various density parameters
Source: Sci Rep. 2021 Jun 24;11:13284. doi: 10.1038/s41598-021-92685-z (PMC8225914; doi:10.1038/s41598-021-92685-z)
Supplement: Supplementary file 1 — Supplementary Information. [file 41598_2021_92685_MOESM1_ESM.pdf]

Influence of osteoporosis on the compressive properties of femoral cancellous bone and its dependence on various density parameters

Supplementary Material

Metzner, F.[1,2,3] *.; Neupetsch, C.[1,2]; Fischer, J.-P.[3,4] Drossel, W.-G.[1,2]; Heyde C.-E.[3,4].; Schleifenbaum, S.[2,3,4]

First and second author contributed equally to this publication

1.) Professorship for Adaptronics and Lightweight Design in Production, Institute for Machine Tools and Production Processes, Chemnitz University of Technology, Chemnitz, Germany

2.) Fraunhofer Institute for Machine Tools and Forming Technology, Dresden, Germany

3.) ZESBO - Center for Research on Musculoskeletal Systems, Department of Orthopaedic Surgery, Traumatology and Plastic Surgery, Leipzig University, Leipzig, Germany

4.) Department of Orthopedic Surgery, Traumatology and Plastic Surgery, Leipzig University, Leipzig, Germany

Corresponding Author

M.Sc. Florian Metzner
Research associate
Phone: +49 341 97 23241
E-Mail: florian.metzner@mb.tu-chemnitz.de
Address: Semmelweisstraße 14, 04103 Leipzig, Germany

1. T-Test Male vs. Female

Group Statistics

	Gender	N	Mean	Std. Deviation	Std. Error Mean
Age	Male	20	80,75	8,693	1,944
	Female	21	80,67	12,963	2,829
T_Score	Male	20	-,99900	1,307476	,292361
	Female	19	-2,40526	,916196	,210190
BMD	Male	20	,79295	,179431	,040122
	Female	19	,59958	,109484	,025117
ρ_b_1	Male	19	1,2901	,09278	,02129
	Female	18	1,2421	,10620	,02503
ρ_b_2	Male	19	1,2899	,18035	,04137
	Female	18	1,2844	,11420	,02692
ρ_r_1	Male	19	70,4572	10,89609	2,49973
	Female	18	64,0048	10,56578	2,49038
ρ_r_2	Male	19	48,4813	16,93503	3,88516
	Female	18	35,2951	9,74101	2,29598
ρ_app_1	Male	19	,9062	,13981	,03207
	Female	18	,7928	,13884	,03273
ρ_app_2	Male	19	,6132	,20508	,04705
	Female	18	,4474	,10863	,02560
σ_p	Male	18	8,83256	3,905320	,920493
	Female	15	8,25413	4,090962	1,056282
σ_max	Male	20	9,67900	7,118355	1,591713
	Female	19	6,92626	3,811073	,874320
E	Male	20	604,63125	261,166803	58,398673
	Female	19	619,87589	293,786402	67,399223

Independent Samples Test

		Levene's Test for Equality of Variances		t-test for Equality of Means						
									95% Confidence Interval of the Difference	
		F	Sig.	t	df	Sig. (2-tailed)	Mean Difference	Std. Error Difference	Lower	Upper
Age	Equal variances assumed	6,232	,017	,024	39	,981	,083	3,465	-6,925	7,092
	Equal variances not assumed			,024	35,107	,981	,083	3,432	-6,884	7,050
T_Score	Equal variances assumed	5,167	,029	3,871	37	,000	1,406263	,363326	,670094	2,142432
	Equal variances not assumed			3,905	34,101	,000	1,406263	,360076	,674581	2,137945
BMD	Equal variances assumed	8,655	,006	4,036	37	,000	,193371	,047909	,096298	,290444
	Equal variances not assumed			4,085	31,675	,000	,193371	,047336	,096913	,289829
ρ_b_1	Equal variances assumed	1,019	,320	1,464	35	,152	,04794	,03274	-,01852	,11440
	Equal variances not assumed			1,459	33,789	,154	,04794	,03286	-,01885	,11473
ρ_b_2	Equal variances assumed	2,107	,156	,109	35	,914	,00545	,04995	-,09595	,10685

	Equal variances not assumed			,110	30,648	,913	,00545	,04936	-,09527	,10617
ρ_r_1	Equal variances assumed	,066	,799	1,827	35	,076	6,45243	3,53157	-,71703	13,62190
	Equal variances not assumed			1,829	34,978	,076	6,45243	3,52855	-,71106	13,61592
ρ_r_2	Equal variances assumed	9,885	,003	2,881	35	,007	13,18615	4,57637	3,89563	22,47668
	Equal variances not assumed			2,922	29,020	,007	13,18615	4,51287	3,95657	22,41573
ρ_app_1	Equal variances assumed	,019	,891	2,473	35	,018	,11332	,04583	,02028	,20637
	Equal variances not assumed			2,473	34,917	,018	,11332	,04582	,02029	,20636
ρ_app_2	Equal variances assumed	5,068	,031	3,048	35	,004	,16582	,05441	,05537	,27627
	Equal variances not assumed			3,096	27,671	,004	,16582	,05356	,05604	,27560
σ_p	Equal variances assumed	,051	,823	,415	31	,681	,578422	1,394995	-2,266690	3,423534
	Equal variances not assumed			,413	29,383	,683	,578422	1,401085	-2,285497	3,442342
σ_max	Equal variances assumed	13,255	,001	1,494	37	,144	2,752737	1,842737	-,981002	6,486476

	Equal variances not assumed			1,516	29,373	,140	2,752737	1,816035	-,959427	6,464900
E	Equal variances assumed	,032	,860	-,171	37	,865	- 15,244645	88,905202	- 195,383695	164,894406
	Equal variances not assumed			-,171	35,967	,865	- 15,244645	89,179932	- 196,115657	165,626368

2. T-Test Left vs. Right

Group Statistics

	Side	N	Mean	Std. Deviation	Std. Error Mean
Age	Left	21	82,10	11,580	2,527
	Right	20	79,25	10,341	2,312
T_Score	Left	20	-1,97000	1,379969	,308571
	Right	19	-1,38316	1,232004	,282641
BMD	Left	20	,65410	,184254	,041200
	Right	19	,74574	,160720	,036872
ρ_b_1	Left	19	1,2716	,09685	,02222
	Right	18	1,2616	,10797	,02545
ρ_b_2	Left	19	1,2773	,13216	,03032
	Right	18	1,2977	,16963	,03998
ρ_r_1	Left	19	66,8303	12,85607	2,94939
	Right	18	67,8332	9,18878	2,16582
ρ_r_2	Left	19	39,2799	16,18350	3,71275
	Right	18	45,0076	14,06921	3,31614
ρ_app_1	Left	19	,8473	,16146	,03704
	Right	18	,8550	,13900	,03276
ρ_app_2	Left	19	,4921	,18402	,04222
	Right	18	,5753	,17793	,04194
σ_p	Left	17	8,03441	3,890195	,943511
	Right	16	9,13831	4,034310	1,008577
σ_max	Left	20	6,90870	4,581238	1,024396
	Right	19	9,84237	6,730502	1,544083
E	Left	20	586,81280	294,922155	65,946599
	Right	19	638,63216	255,281977	58,565702

Independent Samples Test

		Levene's Test for Equality of Variances		t-test for Equality of Means						
									95% Confidence Interval of the Difference	
		F	Sig.	t	df	Sig. (2-tailed)	Mean Difference	Std. Error Difference	Lower	Upper
Age	Equal variances assumed	,320	,575	,828	39	,413	2,845	3,435	-4,102	9,793
	Equal variances not assumed			,831	38,846	,411	2,845	3,425	-4,084	9,774
T_Score	Equal variances assumed	,000	,992	-1,398	37	,170	-,586842	,419698	-1,437231	,263547
	Equal variances not assumed			-1,402	36,865	,169	-,586842	,418452	-1,434811	,261126
BMD	Equal variances assumed	,002	,969	-1,651	37	,107	-,091637	,055488	-,204067	,020793
	Equal variances not assumed			-1,657	36,744	,106	-,091637	,055290	-,203692	,020418
ρ_b_1	Equal variances assumed	,110	,742	,296	35	,769	,00997	,03368	-,05841	,07835
	Equal variances not assumed			,295	34,088	,770	,00997	,03378	-,05868	,07862
ρ_b_2	Equal variances assumed	,007	,935	-,409	35	,685	-,02041	,04984	-,12158	,08077

	Equal variances not assumed			-,407	32,137	,687	-,02041	,05018	-,12260	,08179
ρ_r_1	Equal variances assumed	2,028	,163	-,272	35	,788	-1,00285	3,69226	-8,49853	6,49283
	Equal variances not assumed			-,274	32,607	,786	-1,00285	3,65918	-8,45093	6,44522
ρ_r_2	Equal variances assumed	,522	,475	-1,146	35	,260	-5,72766	4,99737	-15,87286	4,41753
	Equal variances not assumed			-1,151	34,755	,258	-5,72766	4,97808	-15,83626	4,38093
ρ_app_1	Equal variances assumed	,643	,428	-,156	35	,877	-,00774	,04966	-,10854	,09307
	Equal variances not assumed			-,156	34,696	,877	-,00774	,04945	-,10816	,09269
ρ_app_2	Equal variances assumed	,101	,753	-1,397	35	,171	-,08323	,05956	-,20415	,03770
	Equal variances not assumed			-1,399	34,983	,171	-,08323	,05951	-,20404	,03758
σ_p	Equal variances assumed	,059	,810	-,800	31	,430	-1,103901	1,379531	-3,917474	1,709672
	Equal variances not assumed			-,799	30,700	,430	-1,103901	1,381101	-3,921793	1,713991
σ_max	Equal variances assumed	4,429	,042	-1,599	37	,118	-2,933668	1,835176	-6,652089	,784752

	Equal variances not assumed			-1,583	31,543	,123	-2,933668	1,852992	-6,710235	,842898
E	Equal variances assumed	,029	,865	-,585	37	,562	-51,819358	88,531586	-231,201391	127,562675
	Equal variances not assumed			-,588	36,695	,560	-51,819358	88,198046	-230,575703	126,936987

3. T-Test Normal vs. Osteoporosis

Group Statistics

	Osteoporosis_WHO	N	Mean	Std. Deviation	Std. Error Mean
Age	Normal	9	75,89	10,659	3,553
	Osteoporosis	10	88,50	7,397	2,339
T_Score	Normal	9	,26889	,733969	,244656
	Osteoporosis	10	-3,15000	,638140	,201797
BMD	Normal	9	,96567	,104468	,034823
	Osteoporosis	10	,52600	,101056	,031957
ρ_b_1	Normal	8	1,3496	,09379	,03316
	Osteoporosis	9	1,2359	,10045	,03348
ρ_b_2	Normal	8	1,3630	,20743	,07334
	Osteoporosis	9	1,2797	,12287	,04096
ρ_r_1	Normal	8	72,9768	13,99197	4,94691
	Osteoporosis	9	60,0649	6,45287	2,15096
ρ_r_2	Normal	8	59,8340	13,95933	4,93537
	Osteoporosis	9	34,9587	12,59395	4,19798
ρ_app_1	Normal	8	,9808	,17442	,06167
	Osteoporosis	9	,7441	,10723	,03574
ρ_app_2	Normal	8	,7948	,14177	,05012
	Osteoporosis	9	,4382	,13148	,04383
σ_p	Normal	8	11,30050	3,473780	1,228167
	Osteoporosis	8	7,53500	3,892683	1,376271
σ_max	Normal	9	14,11411	7,310808	2,436936
	Osteoporosis	10	6,15700	3,053231	,965516
E	Normal	9	647,24400	299,580037	99,860012
	Osteoporosis	10	604,46910	200,184081	63,303765

Independent Samples Test

		Levene's Test for Equality of Variances		t-test for Equality of Means						
									95% Confidence Interval of the Difference	
		F	Sig.	t	df	Sig. (2-tailed)	Mean Difference	Std. Error Difference	Lower	Upper
Age	Equal variances assumed	,804	,383	-3,023	17	,008	-12,611	4,172	-21,413	-3,810
	Equal variances not assumed			-2,965	14,086	,010	-12,611	4,254	-21,730	-3,493
T_Score	Equal variances assumed	1,264	,277	10,864	17	,000	3,418889	,314694	2,754943	4,082834
	Equal variances not assumed			10,780	16,004	,000	3,418889	,317142	2,746591	4,091187
BMD	Equal variances assumed	,753	,397	9,320	17	,000	,439667	,047176	,340134	,539200
	Equal variances not assumed			9,302	16,651	,000	,439667	,047263	,339790	,539543
ρ_b_1	Equal variances assumed	,013	,910	2,403	15	,030	,11374	,04733	,01286	,21461
	Equal variances not assumed			2,413	14,951	,029	,11374	,04713	,01326	,21421
ρ_b_2	Equal variances assumed	,544	,472	1,022	15	,323	,08333	,08150	-,09038	,25705

	Equal variances not assumed			,992	11,103	,342	,08333	,08400	-,10134	,26801
ρ_r_1	Equal variances assumed	3,380	,086	2,493	15	,025	12,91186	5,17832	1,87453	23,94920
	Equal variances not assumed			2,394	9,597	,039	12,91186	5,39430	,82381	24,99991
ρ_r_2	Equal variances assumed	,220	,646	3,864	15	,002	24,87533	6,43769	11,15373	38,59694
	Equal variances not assumed			3,839	14,261	,002	24,87533	6,47927	11,00253	38,74814
ρ_app_1	Equal variances assumed	2,470	,137	3,416	15	,004	,23664	,06928	,08897	,38431
	Equal variances not assumed			3,320	11,370	,007	,23664	,07128	,08038	,39290
ρ_app_2	Equal variances assumed	,121	,732	5,380	15	,000	,35653	,06627	,21528	,49777
	Equal variances not assumed			5,355	14,420	,000	,35653	,06658	,21412	,49894
σ_p	Equal variances assumed	,024	,880	2,041	14	,061	3,765500	1,844591	-,190754	7,721754
	Equal variances not assumed			2,041	13,822	,061	3,765500	1,844591	-,195530	7,726530
σ_max	Equal variances assumed	7,916	,012	3,157	17	,006	7,957111	2,520268	2,639810	13,274413

	Equal variances not assumed			3,036	10,479	,012	7,957111	2,621236	2,152634	13,761588
E	Equal variances assumed	1,411	,251	,370	17	,716	42,774900	115,736694	-201,408181	286,957981
	Equal variances not assumed			,362	13,748	,723	42,774900	118,234465	-211,249263	296,799063

4. T-Test Normal vs Osteopenia

Group Statistics

	Osteoporosis_WHO	N	Mean	Std. Deviation	Std. Error Mean
Age	Normal	9	75,89	10,659	3,553
	Osteopenia	20	77,95	10,753	2,404
T_Score	Normal	9	,26889	,733969	,244656
	Osteopenia	20	-1,83000	,372898	,083382
BMD	Normal	9	,96567	,104468	,034823
	Osteopenia	20	,66500	,051396	,011493
ρ_b_1	Normal	8	1,3496	,09379	,03316
	Osteopenia	20	1,2475	,09023	,02017
ρ_b_2	Normal	8	1,3630	,20743	,07334
	Osteopenia	20	1,2604	,13070	,02922
ρ_r_1	Normal	8	72,9768	13,99197	4,94691
	Osteopenia	20	68,3188	10,17342	2,27485
ρ_r_2	Normal	8	59,8340	13,95933	4,93537
	Osteopenia	20	38,1578	11,38776	2,54638
ρ_app_1	Normal	8	,9808	,17442	,06167
	Osteopenia	20	,8473	,11597	,02593
ρ_app_2	Normal	8	,7948	,14177	,05012
	Osteopenia	20	,4701	,10876	,02432
σ_p	Normal	8	11,30050	3,473780	1,228167
	Osteopenia	17	7,77141	3,764302	,912977
σ_max	Normal	9	14,11411	7,310808	2,436936
	Osteopenia	20	6,82910	4,548919	1,017169
E	Normal	9	647,24400	299,580037	99,860012
	Osteopenia	20	600,01900	304,228156	68,027484

Independent Samples Test

		Levene's Test for Equality of Variances		t-test for Equality of Means						
		F	Sig.	t	df	Sig. (2-tailed)	Mean Difference	Std. Error Difference	95% Confidence Interval of the Difference Lower	Upper
Age	Equal variances assumed	,013	,909	-,479	27	,636	-2,061	4,305	-10,894	6,772
	Equal variances not assumed			-,480	15,626	,638	-2,061	4,290	-11,173	7,051
T_Score	Equal variances assumed	16,452	,000	10,305	27	,000	2,098889	,203670	1,680994	2,516784
	Equal variances not assumed			8,120	9,910	,000	2,098889	,258475	1,522261	2,675516
BMD	Equal variances assumed	17,264	,000	10,497	27	,000	,300667	,028644	,241895	,359439
	Equal variances not assumed			8,199	9,789	,000	,300667	,036670	,218721	,382612
ρ_b_1	Equal variances assumed	,087	,770	2,678	26	,013	,10218	,03815	,02375	,18060
	Equal variances not assumed			2,632	12,509	,021	,10218	,03882	,01798	,18637

ρ_b_2	Equal variances assumed	,838	,368	1,582	26	,126	,10265	,06490	-,03075	,23605
	Equal variances not assumed			1,300	9,313	,225	,10265	,07895	-,07503	,28033
ρ_r_1	Equal variances assumed	1,113	,301	,983	26	,335	4,65800	4,73919	-5,08354	14,39954
	Equal variances not assumed			,855	10,107	,412	4,65800	5,44489	-7,45659	16,77259
ρ_r_2	Equal variances assumed	1,414	,245	4,270	26	,000	21,67620	5,07595	11,24244	32,10996
	Equal variances not assumed			3,903	10,937	,002	21,67620	5,55355	9,44437	33,90803
ρ_app_1	Equal variances assumed	2,951	,098	2,377	26	,025	,13350	,05615	,01808	,24892
	Equal variances not assumed			1,996	9,584	,075	,13350	,06690	-,01644	,28344
ρ_app_2	Equal variances assumed	1,428	,243	6,546	26	,000	,32465	,04960	,22271	,42659
	Equal variances not assumed			5,827	10,470	,000	,32465	,05571	,20127	,44803

σ_p	Equal variances assumed	,007	,934	2,238	23	,035	3,529088	1,577063	,266684	6,791492
	Equal variances not assumed			2,306	14,885	,036	3,529088	1,530334	,265068	6,793109
σ_max	Equal variances assumed	4,042	,054	3,292	27	,003	7,285011	2,213017	2,744276	11,825746
	Equal variances not assumed			2,759	10,891	,019	7,285011	2,640699	1,465778	13,104245
E	Equal variances assumed	,056	,814	,388	27	,701	47,225000	121,563258	-202,202202	296,652202
	Equal variances not assumed			,391	15,722	,701	47,225000	120,829469	-209,290132	303,740132

5. T-Test Osteopenia vs. Osteoporosis

Group Statistics

	Osteoporosis_WHO	N	Mean	Std. Deviation	Std. Error Mean
Age	Osteopenia	20	77,95	10,753	2,404
	Osteoporosis	10	88,50	7,397	2,339
T_Score	Osteopenia	20	-1,83000	,372898	,083382
	Osteoporosis	10	-3,15000	,638140	,201797
BMD	Osteopenia	20	,66500	,051396	,011493
	Osteoporosis	10	,52600	,101056	,031957
ρ_b_1	Osteopenia	20	1,2475	,09023	,02017
	Osteoporosis	9	1,2359	,10045	,03348
ρ_b_2	Osteopenia	20	1,2604	,13070	,02922
	Osteoporosis	9	1,2797	,12287	,04096
ρ_r_1	Osteopenia	20	68,3188	10,17342	2,27485
	Osteoporosis	9	60,0649	6,45287	2,15096
ρ_r_2	Osteopenia	20	38,1578	11,38776	2,54638
	Osteoporosis	9	34,9587	12,59395	4,19798
ρ_app_1	Osteopenia	20	,8473	,11597	,02593
	Osteoporosis	9	,7441	,10723	,03574
ρ_app_2	Osteopenia	20	,4701	,10876	,02432
	Osteoporosis	9	,4382	,13148	,04383
σ_p	Osteopenia	17	7,77141	3,764302	,912977
	Osteoporosis	8	7,53500	3,892683	1,376271
σ_max	Osteopenia	20	6,82910	4,548919	1,017169
	Osteoporosis	10	6,15700	3,053231	,965516
E	Osteopenia	20	600,01900	304,228156	68,027484
	Osteoporosis	10	604,46910	200,184081	63,303765

Independent Samples Test

		Levene's Test for Equality of Variances		t-test for Equality of Means						
						Sig. (2-tailed)	Mean Difference	Std. Error Difference	95% Confidence Interval of the Difference	
		F	Sig.	t	df				Lower	Upper
Age	Equal variances assumed	1,217	,279	-2,779	28	,010	-10,550	3,796	-18,325	-2,775
	Equal variances not assumed			-3,145	24,898	,004	-10,550	3,355	-17,460	-3,640
T_Score	Equal variances assumed	3,138	,087	7,181	28	,000	1,320000	,183814	,943475	1,696525
	Equal variances not assumed			6,045	12,168	,000	1,320000	,218346	,844991	1,795009
BMD	Equal variances assumed	3,191	,085	5,038	28	,000	,139000	,027591	,082483	,195517
	Equal variances not assumed			4,093	11,388	,002	,139000	,033960	,064564	,213436
ρ_b_1	Equal variances assumed	,018	,895	,308	27	,760	,01156	,03748	-,06534	,08846
	Equal variances not assumed			,296	14,082	,772	,01156	,03909	-,07224	,09536
ρ_b_2	Equal variances assumed	,000	,997	-,375	27	,711	-,01932	,05155	-,12509	,08645

	Equal variances not assumed			-,384	16,427	,706	-,01932	,05032	-,12576	,08712
ρ_r_1	Equal variances assumed	,910	,349	2,228	27	,034	8,25386	3,70430	,65327	15,85445
	Equal variances not assumed			2,636	23,517	,015	8,25386	3,13074	1,78530	14,72242
ρ_r_2	Equal variances assumed	,620	,438	,678	27	,504	3,19913	4,71952	-6,48453	12,88280
	Equal variances not assumed			,652	14,163	,525	3,19913	4,90990	-7,32023	13,71849
ρ_app_1	Equal variances assumed	,044	,836	2,265	27	,032	,10314	,04554	,00971	,19657
	Equal variances not assumed			2,336	16,690	,032	,10314	,04416	,00984	,19644
ρ_app_2	Equal variances assumed	,657	,425	,685	27	,499	,03188	,04654	-,06362	,12738
	Equal variances not assumed			,636	13,160	,536	,03188	,05012	-,07627	,14002
σ_p	Equal variances assumed	,007	,936	,145	23	,886	,236412	1,630880	-3,137321	3,610144
	Equal variances not assumed			,143	13,383	,888	,236412	1,651560	-3,321225	3,794049
σ_max	Equal variances assumed	1,728	,199	,420	28	,677	,672100	1,598650	-2,602586	3,946786

	Equal variances not assumed			,479	25,301	,636	,672100	1,402446	-2,214551	3,558751
E	Equal variances assumed	2,648	,115	-,042	28	,967	-4,450100	106,549879	-222,707633	213,807433
	Equal variances not assumed			-,048	25,611	,962	-4,450100	92,925267	-195,602159	186,701959

Regression analysis Archimedean density parameter vs. clinical density parameter

Model Summary[b]

Model	R	R Square	Adjusted R Square	Std. Error of the Estimate
1	,427[a]	,182	,159	,09273

a. Predictors: (Constant), T_Score

b. Dependent Variable: ρ_b_1

Model Summary[b]

Model	R	R Square	Adjusted R Square	Std. Error of the Estimate
1	,427[a]	,182	,159	,09273

a. Predictors: (Constant), T_Score

b. Dependent Variable: ρ_b_1

Model Summary[b]

Model	R	R Square	Adjusted R Square	Std. Error of the Estimate
1	,261[a]	,068	,042	,14662

a. Predictors: (Constant), T_Score

b. Dependent Variable: ρ_b_2

Model Summary[b]

Model	R	R Square	Adjusted R Square	Std. Error of the Estimate
1	,324[a]	,105	,079	10,63079

a. Predictors: (Constant), T_Score

b. Dependent Variable: ρ_r_1

Model Summary[b]

Model	R	R Square	Adjusted R Square	Std. Error of the Estimate
1	,572[a]	,327	,308	12,69269

a. Predictors: (Constant), T_Score

b. Dependent Variable: ρ_r_2

Model Summary[b]

Model	R	R Square	Adjusted R Square	Std. Error of the Estimate
1	,494[a]	,244	,222	,13134

a. Predictors: (Constant), T_Score

b. Dependent Variable: ρ_app_1

Model Summary[b]

Model	R	R Square	Adjusted R Square	Std. Error of the Estimate
1	,702[a]	,493	,478	,13253

a. Predictors: (Constant), T_Score

b. Dependent Variable: ρ_app_2

Model Summary[b]

Model	R	R Square	Adjusted R Square	Std. Error of the Estimate
1	,400[a]	,160	,136	,09396

a. Predictors: (Constant), BMD

b. Dependent Variable: ρ_b_1

Model Summary[b]

Model	R	R Square	Adjusted R Square	Std. Error of the Estimate
1	,209[a]	,044	,016	,14854

a. Predictors: (Constant), BMD

b. Dependent Variable: ρ_b_2

Model Summary[b]

Model	R	R Square	Adjusted R Square	Std. Error of the Estimate
1	,286[a]	,082	,056	10,76813

a. Predictors: (Constant), BMD

b. Dependent Variable: ρ_r_1

Model Summary[b]

Model	R	R Square	Adjusted R Square	Std. Error of the Estimate
1	,620[a]	,384	,366	12,14803

a. Predictors: (Constant), BMD

b. Dependent Variable: ρ_r_2

Model Summary[b]

Model	R	R Square	Adjusted R Square	Std. Error of the Estimate
1	,450[a]	,203	,180	,13483

a. Predictors: (Constant), BMD

b. Dependent Variable: ρ_app_1

Model Summary[b]

Model	R	R Square	Adjusted R Square	Std. Error of the Estimate
1	,737[a]	,543	,530	,12584

a. Predictors: (Constant), BMD

b. Dependent Variable: ρ_app_2

Regression analysis mechanical parameter vs. density parameter

Model Summary and Parameter Estimates

Dependent Variable: E

	Model Summary					Parameter Estimates	
Equation	R Square	F	df1	df2	Sig.	Constant	b1
Linear	,047	1,714	1	35	,199	-101,105	572,312
Power	,033	1,210	1	35	,279	414,592	1,250
Exponential	,032	1,158	1	35	,289	165,600	,955

The independent variable is ρ_b_1.

Model Summary and Parameter Estimates

Dependent Variable: E

	Model Summary					Parameter Estimates	
Equation	R Square	F	df1	df2	Sig.	Constant	b1
Linear	,108	4,229	1	35	,047	-131,805	587,041
Power	,069	2,577	1	35	,117	407,009	1,260
Exponential	,076	2,884	1	35	,098	154,442	,994

The independent variable is ρ_b_2.

Model Summary and Parameter Estimates

Dependent Variable: E

	Model Summary					Parameter Estimates	
Equation	R Square	F	df1	df2	Sig.	Constant	b1
Linear	,010	,347	1	35	,560	785,077	-2,395
Power	,005	,188	1	35	,667	1499,520	-,237
Exponential	,004	,144	1	35	,707	684,449	-,003

The independent variable is ρ_r_1.

Model Summary and Parameter Estimates

Dependent Variable: E

	Model Summary					Parameter Estimates	
Equation	R Square	F	df1	df2	Sig.	Constant	b1
Linear	,133	5,383	1	35	,026	354,359	6,407
Power	,226	10,239	1	35	,003	44,203	,689
Exponential	,183	7,849	1	35	,008	293,709	,015

The independent variable is ρ_r_2.

Model Summary and Parameter Estimates

Dependent Variable: E

Equation	Model Summary					Parameter Estimates	
	R Square	F	df1	df2	Sig.	Constant	b1
Linear	,000	,000	1	35	,998	623,201	,774
Power	,000	,010	1	35	,920	560,315	,054
Exponential	,000	,010	1	35	,920	526,528	,062

The independent variable is ρ_app_1.

Model Summary and Parameter Estimates

Dependent Variable: E

Equation	Model Summary					Parameter Estimates	
	R Square	F	df1	df2	Sig.	Constant	b1
Linear	,227	10,264	1	35	,003	253,768	694,954
Power	,360	19,714	1	35	,000	1058,483	,940
Exponential	,271	13,023	1	35	,001	245,592	1,531

The independent variable is ρ_app_2.

Model Summary and Parameter Estimates

Dependent Variable: σ_max

Equation	Model Summary					Parameter Estimates	
	R Square	F	df1	df2	Sig.	Constant	b1
Linear	,191	8,237	1	35	,007	-23,647	25,377
Power	,127	5,095	1	35	,030	2,877	3,518
Exponential	,128	5,149	1	35	,030	,199	2,758

The independent variable is ρ_b_1.

Model Summary and Parameter Estimates

Dependent Variable: σ_max

Equation	Model Summary					Parameter Estimates	
	R Square	F	df1	df2	Sig.	Constant	b1
Linear	,153	6,319	1	35	,017	-11,258	15,348
Power	,090	3,452	1	35	,072	3,918	2,080
Exponential	,101	3,915	1	35	,056	,783	1,649

The independent variable is ρ_b_2.

Model Summary and Parameter Estimates

Dependent Variable: σ_max

Equation	Model Summary					Parameter Estimates	
	R Square	F	df1	df2	Sig.	Constant	b1
Linear	,007	,237	1	35	,629	5,567	,044
Power	,002	,072	1	35	,790	2,685	,212
Exponential	,004	,155	1	35	,696	4,777	,005

The independent variable is ρ_r_1.

Model Summary and Parameter Estimates

Dependent Variable: σ_max

Equation	Model Summary					Parameter Estimates	
	R Square	F	df1	df2	Sig.	Constant	b1
Linear	,292	14,420	1	35	,001	-,254	,208
Power	,392	22,589	1	35	,000	,053	1,309
Exponential	,343	18,293	1	35	,000	1,859	,030

The independent variable is ρ_r_2.

Model Summary and Parameter Estimates

Dependent Variable: σ_max

Equation	Model Summary					Parameter Estimates	
	R Square	F	df1	df2	Sig.	Constant	b1
Linear	,074	2,811	1	35	,103	-,661	10,763
Power	,046	1,682	1	35	,203	7,766	,979
Exponential	,049	1,822	1	35	,186	2,431	1,163

The independent variable is ρ_app_1.

Model Summary and Parameter Estimates

Dependent Variable: σ_max

Equation	Model Summary					Parameter Estimates	
	R Square	F	df1	df2	Sig.	Constant	b1
Linear	,507	35,963	1	35	,000	-3,647	22,807
Power	,598	51,963	1	35	,000	21,710	1,747
Exponential	,522	38,230	1	35	,000	1,278	3,066

The independent variable is ρ_app_2.

Model Summary and Parameter Estimates

Dependent Variable: σ_p

Equation	Model Summary					Parameter Estimates	
	R Square	F	df1	df2	Sig.	Constant	b1
Linear	,078	2,527	1	30	,122	-5,165	10,757
Power	,041	1,295	1	30	,264	4,401	1,982
Exponential	,038	1,179	1	30	,286	1,079	1,475

The independent variable is ρ_b_1.

Model Summary and Parameter Estimates

Dependent Variable: σ_p

Equation	Model Summary					Parameter Estimates	
	R Square	F	df1	df2	Sig.	Constant	b1
Linear	,056	1,782	1	30	,192	,718	6,041
Power	,048	1,504	1	30	,230	4,884	1,466
Exponential	,049	1,546	1	30	,223	1,678	1,110

The independent variable is ρ_b_2.

Model Summary and Parameter Estimates

Dependent Variable: σ_p

Equation	Model Summary					Parameter Estimates	
	R Square	F	df1	df2	Sig.	Constant	b1
Linear	,115	3,903	1	30	,057	,516	,119
Power	,114	3,865	1	30	,059	,011	1,551
Exponential	,118	4,016	1	30	,054	1,430	,024

The independent variable is ρ_r_1.

Model Summary and Parameter Estimates

Dependent Variable: σ_p

Equation	Model Summary					Parameter Estimates	
	R Square	F	df1	df2	Sig.	Constant	b1
Linear	,134	4,657	1	30	,039	4,184	,099
Power	,079	2,575	1	30	,119	,638	,644
Exponential	,094	3,121	1	30	,087	3,450	,016

The independent variable is ρ_r_2.

Model Summary and Parameter Estimates

Dependent Variable: σ_p

Equation	Model Summary					Parameter Estimates	
	R Square	F	df1	df2	Sig.	Constant	b1
Linear	,229	8,921	1	30	,006	-2,447	12,834
Power	,198	7,416	1	30	,011	10,025	2,067
Exponential	,181	6,629	1	30	,015	1,037	2,241

The independent variable is ρ_app_1.

Model Summary and Parameter Estimates

Dependent Variable: σ_p

Equation	Model Summary					Parameter Estimates	
	R Square	F	df1	df2	Sig.	Constant	b1
Linear	,228	8,876	1	30	,006	2,382	10,943
Power	,161	5,742	1	30	,023	13,497	1,045
Exponential	,156	5,532	1	30	,025	2,601	1,776

The independent variable is ρ_app_2.

Model Summary and Parameter Estimates

Dependent Variable: E

Equation	Model Summary					Parameter Estimates	
	R Square	F	df1	df2	Sig.	Constant	b1
Linear	,023	,860	1	37	,360	449,194	233,081
Power	,009	,345	1	37	,561	583,638	,227
Exponential	,011	,413	1	37	,524	417,965	,351

The independent variable is BMD.

Model Summary and Parameter Estimates

Dependent Variable: σ_max

Equation	Model Summary					Parameter Estimates	
	R Square	F	df1	df2	Sig.	Constant	b1
Linear	,242	11,800	1	37	,001	-3,001	16,228
Power	,099	4,077	1	37	,051	9,383	1,018
Exponential	,113	4,705	1	37	,037	2,158	1,537

The independent variable is BMD.

Model Summary and Parameter Estimates

Dependent Variable: σ_p

Equation	Model Summary					Parameter Estimates	
	R Square	F	df1	df2	Sig.	Constant	b1
Linear	,105	3,649	1	31	,065	3,528	7,126
Power	,042	1,372	1	31	,250	9,082	,642
Exponential	,044	1,422	1	31	,242	3,761	,905

The independent variable is BMD.
